# Gabapentin as add-on to morphine for severe neuropathic or mixed pain in children from age 3 months to 18 years - evaluation of the safety, pharmacokinetics, and efficacy of a new gabapentin liquid formulation: study protocol for a randomized controlled trial

**DOI:** 10.1186/s13063-018-3169-3

**Published:** 2019-01-15

**Authors:** Thomas G. de Leeuw, Laura Mangiarini, Rebecca Lundin, Florentia Kaguelidou, Tjitske van der Zanden, Oscar Della Pasqua, Dick Tibboel, Adriana Ceci, Saskia N. de Wildt, on behalf of the GAPP consortium, Donjeta Bali, Alketa Hoxha-Qosja, Ermira Kola, Juliette Andrieu-Galien, Daniel Annequin, Romy Blanchet, Isabelle Desguerre, Elisabeth Fournier-Charrière, Florentia Kaguelidou, Barbara Tourniaire, Chantal Wood, Antje Neubert, Regina Trollmann, Stefan Wimmer, Eleana Garini, Panagoula Mammi, Marcello Allegretti, Ornella Bellagamba, Franca Benini, Donato Bonifazi, Daniela Caprino, Adriana Ceci, Sabrina Congedi, Francesco Craig, Sandro Dallorso, Antuan Divisic, Mariagrazia Felisi, Marco Gentile, Andrea De Giacomo, Rebecca Lundin, Luca Manfredini, Laura Mangiarini, Emilia Matera, Lucia Margari, Alessandro Mazza, Virgilio Pace, Chiara Di Pede, Maria Giuseppina Petruzzelli, Pieradelchi Ruffini, Luigina Tagliavacca, Maria Traverso, Anna Szumowska, Tom de Leeuw, Maarten Mensink, Lonneke Staals, Dick Tibboel, Saskia N. de Wildt, Tjitske van der Zanden, Dmytro Delva, Andrii Loboda, Katerina Savinova, Grygorii Ursol, Helen Neary, Oscar Della Pasqua, Paul Healy

**Affiliations:** 1 Department of Anesthesia and Pain Medicine, Erasmus MC-Sophia Children’s Hospital, Dr. Molewaterplein 40, 3015 GD Rotterdam, The Netherlands; 2 Consorzio per Valutazione Biologiche e Farmacologiche, Pavia, Italy; 3 Fondazione Penta Onlus, Padova, Italy; 4 Department of Pediatric Pharmacology and Pharmacogenetics, AP-HP, Hôpital Robert Debré, F-75019 Paris, France; 5 Inserm, CIC 1426, F-75019, Paris, France; 6 Université Paris Diderot, Sorbonne Paris Cité, EA 08, F-75010 Paris, France; 7 Intensive Care and Department of Paediatric Surgery, Erasmus MC-Sophia Children’s Hospital, Dr. Molewaterplein 40, 3015 GD Rotterdam, The Netherlands; 8 University College London, London, UK; 9 Department of Pharmacology and Toxicology, Radboud University, Nijmegen, The Netherlands

**Keywords:** Gabapentin, Neuropathic pain, Children, Pharmacokinetics

## Abstract

**Background:**

Gabapentin has shown efficacy in the treatment of chronic neuropathic or mixed pain in adults. Although pediatric pain specialists have extensive experience with gabapentin for the treatment of neuropathic pain, its use is off-label. Its efficacy and safety in this context have never been shown. The aim of this trial is to compare gabapentin with placebo as add-on to morphine for the treatment of severe chronic mixed or neuropathic pain in children. This trial is part of the European Union Seventh Framework Programme project Gabapentin in Paediatric Pain (GAPP) to develop a pediatric use marketing authorization for a new gabapentin suspension.

**Methods/design:**

The GAPP-2 study is a randomized, double-blind, placebo-controlled, multicenter superiority phase II study in children with severe chronic neuropathic or mixed pain. Its primary objective is to evaluate the efficacy of a gabapentin liquid formulation as adjunctive therapy to morphine. Sixty-six eligible children 3 months to 18 years of age with severe pain (pain scores ≥ 7), stratified in three age groups, will be randomized to receive gabapentin (to an accumulating dose of 45 to 63 mg/kg/day, dependent on age) or placebo, both in addition to morphine, for 12 weeks. Randomization will be preceded by a short washout period, and treatment will be initiated by a titration period of 3 weeks. After the treatment period, medication will be tapered during 4 weeks. The primary endpoint is the average pain scores in the two treatment groups (average of two measures each day for 3 days before the end-of-study visit [V10] assessed by age-appropriate pain scales (Face, Legs, Activity, Cry, Consolability scale; Faces Pain Scale–Revised; Numeric Rating Scale). Secondary outcomes include percentage responders to treatment (subjects with 30% reduction in pain scale), number of episodes of breakthrough pain, number of rescue interventions, number of pain-free days, participant dropouts, quality of life (Pediatric Quality of Life Inventory), and acceptability of treatment. Outcomes will be measured at the end-of-study visit after 12 weeks of treatment at the optimal gabapentin dose. Groups will be compared on an intention-to-treat basis.

**Discussion:**

We hope to provide evidence that the combination of morphine and gabapentin will provide better analgesia than morphine alone and will be safe. We also aim to obtain confirmation of the recommended pediatric dose.

**Trial registration:**

EudractCT, 2014-004897-40. Registered on 7 September 2017.

ClinicalTrials.gov, NCT03275012. Registered on 7 September 2017.

**Electronic supplementary material:**

The online version of this article (10.1186/s13063-018-3169-3) contains supplementary material, which is available to authorized users.

## Background

Chronic pain, continuous or recurrent, lasting for more than 3 months affects 11–35% of children with varying disabilities [1, 2]. Although nonsteroidal anti-inflammatory drugs and opioids may be the standard therapy for mild and severe non-neuropathic pain (non-NP) in both adults and children, and although tramadol and morphine have shown efficacy in different types of NP in adults [3–5], they are not considered to be the first-line medications for NP syndromes [6–8], owing to concerns regarding long-term safety. Nevertheless, opioids are used for patients with severe NP not responding to first-line medications in acute NP or episodic exacerbation of severe NP. Because of a lack of appropriate studies in children concerning management of chronic NP, the latter are often undertreated or pain medication is used off-label.

The antiepileptic drug gabapentin has shown efficacy in a wide range of neuropathic or mixed pain syndromes. The mechanism of gabapentin is at the voltage-activated calcium channels in the central nervous system (CNS), but the mode of action in the treatment of NP is still not fully understood. In early studies, gabapentin has been shown to have a central antiallodynic effect [9] as well as to inhibit ectopic discharge activity from injured peripheral nerves [10]. Although originally the hypothesis was that it exerted its antiallodynic effect through γ-aminobutyric acid-mediated pathways at the spinal cord and brain level or by antagonism of *N*-methyl-d-aspartate receptors, evidence is emerging for antagonism of calcium channels in the CNS and peripheral nerves [9, 11]. Fink et al. showed that gabapentin in the neocortex of the rat but also in humans inhibits neuronal calcium influx, leading to decreased α-amino-3-hydroxy-5-methyl-4-isoxazole propionic acid receptor activation and noradrenaline release in the brain [12]. More recently, it was suggested that gabapentin also inhibits activation of a nuclear transcription factor and consequently expression of cyclooxygenase 2 and other genes involved in inflammation [13].

In adults, randomized controlled trials of gabapentin have shown its efficacy and safety for the treatment of postherpetic neuralgia [14, 15], diabetic neuropathy [16], phantom limb pain [17], spinal cord injury [18], peripheral nerve injury [19], and neuropathic cancer pain [20]. Case series and case reports further suggest its efficacy for analgesia in multiple sclerosis [21, 22], complex regional pain syndrome type 1, erythromelalgia, trigeminal neuralgia, peripheral neuropathy, post-thoracotomy neuropathy, central pain syndromes, and Guillain-Barré syndrome [23].

Efficacy in pediatric pain is based mainly on anecdotal reports and several open-label, noncontrolled clinical studies. Gabapentin appears efficacious for the treatment of post-thoracotomy pain, complex regional pain syndrome type 1, phantom limb pain, and spinal fusion surgery [24–26].

Hence, although pediatric pain specialists have extensive experience with the use of gabapentin and reports support its benefits, efficacy and safety have not been unequivocally demonstrated in well-designed clinical efficacy and safety studies in the pediatric population.

In this context, the GAPP (Gabapentin in Paediatric Pain) project is a European-funded project that comprises a full pediatric development program for gabapentin in the treatment of chronic neuropathic or mixed pain in children. The development strategy, requirements, and regulatory deliverables have been outlined in a pediatric investigation plan (PIP), which has agreed with and approved by the European Medicines Agency’s Paediatric Committee.

The PIP includes (1) the development of a liquid oral gabapentin formulation, (2) the evaluation of gabapentin safety in juvenile animal toxicity studies (PRE-GABA), (3) two clinical trials to evaluate the efficacy and safety of gabapentin as monotherapy (GABA-1) and as adjuvant therapy (GABA-2), and (4) a modeling bridging study (GABA-3) to specifically address the paucity of pharmacokinetic (PK) data in children and enhance the dose rationale for the pediatric population [27]. The study protocol presented in this paper concerns the GABA-2 trial. The primary aim of this specific study (GABA-2) is to determine the efficacy and safety of gabapentin versus placebo as add-on to morphine in children with severe chronic neuropathic or mixed pain.

## Methods/design

In this randomized, double-blind, placebo controlled, multicenter superiority phase II study, the efficacy of gabapentin as add-on to morphine will be compared with the efficacy and safety of placebo as add-on to morphine.

### Study population

A total of 66 children from 3 months to 18 years of age with chronic (> 3 months) neuropathic or mixed (neuropathic and nociceptive component) pain are being recruited into the trial. Subjects are recruited in several centers for pediatric pain divided over six European Union countries (Table 1).

**Table 1:** List of recruiting centers participating in the GABA-2 clinical trial

Albania	Qendra Spitalore Universitare Nene Tereza General Pediatric Clinic - Pediatric Department Rruga e Dibrës 372, 1000 Tiranë, Albania Principal Investigator: Prof. Ermira Kola
France	Assistance Publique Hôpitaux de Paris - APHP Hôpital Robert Debré Centre of Clinical Investigations, INSERM CIC1426 Boulevard Sérurier 48, 75,019, Paris, France Principal Investigator (Country Coordinator): Dr. Florentia Kaguelidou Centre d’évaluation et de traitement de la douleur Co-investigator: Dr. Sophie Dugué
	Assistance Publique Hôpitaux de Paris - APHP Hôpital Necker Centre d’évaluation et de traitement de la douleur Rue de Sèvres 149, 75,015, Paris, France Principal Investigator: Dr. Brigitte Charron
	Hôpital d’enfants Armand Trousseau* Centre de Référence de la migraine de l’enfant et de l’adolescent et du Centre de la douleur 26 avenue du Docteur Arnold-Netter, 75,012 Paris Sub-Investigator: Prof. Barbara Tournaire
	Assistance Publique Hôpitaux de Marseille - APHM Hôpital La Timone Service de Pédiatrie Rue Saint-Pierre 264, 13,005, Marseille, France Principal Investigator: Dr. Cécile Mareau
	Centre Hospitalier Régional Universitaire de Lille - CHRU Lille Hôpital Roger Salengro Service de Neuropédiatrie - Consultation Douleur Enfant Rue Emile Laine, 59,037, Lille, France Principal Investigator: Dr. Justine Avez-Couturier
Germany	Universitaetsklinikum Erlangen Department of Paediatrics and Adolescent Medicine Loschgestraße 15, D-91054 Erlangen, Germany Principal Investigator: Prof. Regina Trollmann
Greece	Geniko Nosokomeio Paidon I Agia Sofia Anaesthetic department & Pain Clinic Thivon & Papadiamantopoulou 1, 11,527 Athens, Greece Principal Investigator: Dr. Panagoula Mammi
Italy	Azienda Ospedaliero - Universitaria Consorziale Policlinico di Bari U.O.C. di Neuropsichiatria Infantile Piazza Giulio Cesare 11, 70,124, Bari, Italy Principal Investigator (Country Coordinator): Prof. Lucia Margari
	Azienda Ospedaliera di Padova Department of Women and Child Health Via Giustiniani 2, 35,128, Padova, Italy Principal Investigator: Prof. Franca Benini
	Istituto Giannina Gaslini – Genova Unità Operativa Semplice Dipartimentale di Assistenza domiciliare e Continuità delle Cure Dipartimento Testa - Collo e Neuroscienze Via Gerolamo Gaslini 5, 16,148, Genova, Italy Principal Investigator: Dr. Luca Manfredini
The Netherlands	Erasmus Universitair Medisch Centrum Rotterdam - Sophia Kinderziekenhuis Intensive Care and Department of Paediatric Surgery Department of Anesthesiology Wijtemaweg 80, 3015 CN, Rotterdam, the Netherlands Principal Investigator (Country Coordinator): Prof. Saskia N. De Wildt
	University Medical Center Utrecht, Wilhelmina Kinderziekenhuis Department of Anesthesiology Heidelberglaan 100, 3584 CX, Utrecht, The Netherlands Principal Investigator: Dr. Maarten O. Mensink

#### Inclusion criteria

Subjects must fulfill the following inclusion criteria to be eligible:

1. Children aged 3 months to 18 years
2. Diagnosis of neuropathic or mixed pain

Because there are no validated tools for the diagnosis of NP in children, diagnosis will be based on Treede et al. criteria [28]:

- Pain with a distinct neuroanatomically plausible distribution
- Medical history suggestive of a relevant lesion or disease affecting peripheral or central somatosensory system
- Clinical examination with demonstration of a distinct neuroanatomical distribution by at least one confirmatory test
- Diagnostic test confirming lesion or underlying disease (e.g., magnetic resonance imaging or computed tomographic scan or laboratory test confirming metabolic disease)

Patients must at least meet one of four mentioned criteria if younger than 3 years old and at least two of four criteria if older than 3 years of age [28].

For complex regional pain syndrome, the so-called Budapest criteria will be used for the diagnosis [29]. This means there is continuing pain disproportionate to the inciting event.

Patient must report at least one symptom in three of four of the following categories:

- Sensory: allodynia and/or hyperalgesia
- Vasomotor: temperature asymmetry, skin color changes/asymmetry
- Pseudomotor/edema: edema and/or sweating
- Motor/trophic: motor dysfunction and/or trophic changes (nail/skin)

This together with at least one sign during evaluation in two or more of the previous categories and no other diagnosis can explain the symptoms.

3. Severe pain

Severe pain is defined as intensity 7 or more assessed during a 3-day screening period using the following scores according to age: Face, Legs, Activity, Cry, Consolability scale (FLACC) for children ages 3 months up to and including 2 years; Faces Pain Scale–Revised (FPS-R) for children aged 3 years up to and including 7 years; and the pain Numeric Rating Scale (NRS-11) for children between 8 and 18 years old. Pain intensity is assessed two times daily and at least five of six assessments should be available.

4. There should be chronic or recurrent pain for a period of at least 3 months.
5. Informed consent from parents or legal guardian
6. A stable underlying disease condition and treatment
7. Patients with chemotherapy-induced peripheral neuropathy when in clinical remission or maintenance phase of their therapeutic protocol

#### Exclusion criteria

1. Pain duration of more than 5 years
2. Current use of gabapentin
3. Current use of strong opioids (morphine, methadone, fentanyl, oxycodone) or ketamine
4. History of failure to respond to treatment with gabapentin or opioids for NP
5. History of epilepsy (except febrile seizure)
6. Subjects diagnosed with sickle cell disease
7. Subjects diagnosed with cognitive impairment
8. Subjects who present with current controlled or uncontrolled comorbid psychiatric diagnosis that can impair pain diagnosis and assessment
9. Subjects with a history of or current suicidal ideation or behavior
10. Subjects with a history of substance abuse in particular opioids
11. Subjects being treated with prohibited concomitant medication (*see* Table 2)
12. Subjects with a body mass index below the 5th percentile or above the 95th percentile for their age and gender
13. Subjects with renal impairment (i.e., glomerular filtration rate < 90 ml/min/1.73 m^2^)
14. Subjects with hepatic impairment (aspartate transaminase/aAlanine transaminase three times the upper limit of normal for age)
15. Corticosteroid oral treatment or infiltration needed for pain caused by infiltration or compression of neural structures
16. Subjects with clinically relevant abnormal electrocardiogram (ECG) at the screening visit
17. Subjects with a known allergy/hypersensibility/significant intolerance to any component of the study drugs
18. Subjects with fructose intolerance, diabetes, glucose-galactose malabsorption, or lactase-isomaltase deficiency
19. Subjects participating in another clinical trial
20. Subjects scheduled for surgery or in recovery from surgery within 3 months of baseline assessment
21. Female subjects who are pregnant or lactating
22. Subjects who fail screening or were previously enrolled in this study
23. Patients with chemotherapy-induced peripheral neuropathy when in induction phase of their therapeutic protocol

**Table 2:** Prohibited concomitant medication

- Tricyclic antidepressants, selective serotonin reuptake inhibitors, selective noradrenalin reuptake inhibitors, antipsychotics, neuroleptics, anxiolytics, benzodiazepines, psychostimulants, mono amine oxidase inhibitors, sedatives	
- Anticonvulsant medications as pregabalin, valproic acid, etc.	
- All NSAIDs with exception of ibuprofen	
- Opioids	
- Benzodiazepines	
- Ketamine	
- Lidocaine	
- Medical cannabis	

*NSAIDs* Nonsteroidal anti-inflammatory drugs

### Screening, assessment, and randomization

#### Screening period

After obtaining informed consent from the subjects’ parents or legal representatives, during a screening period to confirm study eligibility, a medical history, including all relevant lifetime medication and nonpharmacological interventions, will be obtained, and concomitant medication will be recorded. Also, required clinical laboratory tests will be done. Venous blood samples will be taken for standard clinical hematology and biochemistry (2.5 ml) together with a serum β-human chorionic gonadotropin test (in females of childbearing age) (2 ml) and a sample for pharmacogenomics (0.5 ml) and metabolomics (1 ml).

A washout period of 3 days may be required to discontinue medication potentially interfering with the primary outcome of pain intensity, with the exception of the rescue medications paracetamol and ibuprofen.

#### Baseline assessment

After the washout period, the patient and, dependent on the age of the child, the child’s parents will be requested to assess pain intensity twice daily for 3 days to obtain the average baseline pain intensity. During this period, rescue medication will be allowed (i.e., paracetamol 15 mg/kg [oral or rectal] four to six times daily to a maximum daily dose of 4 g or ibuprofen 5–10 mg/kg [oral] every 6–8 h to a maximum daily dose of 30 mg/kg/day).

#### Randomization

After the screening period, the patients return to the trial center and will be randomized (on V2) at a 1:1 ratio to one of the treatment groups: gabapentin plus morphine or placebo plus morphine.

Randomization is performed using ICE version 1.0 software and generated by a statistician not involved in data analysis of trial results. After randomization login, a blinded message is sent to the investigator, and an unblinded message is sent to the pharmacy.

Patients are stratified into three age groups (3 months to 3 years, 3 years to 8 years, and 8 years to 18 years) with pain scores validated for that age group.

### Treatment period

#### Dose and dose optimization

**Fig. 1:**
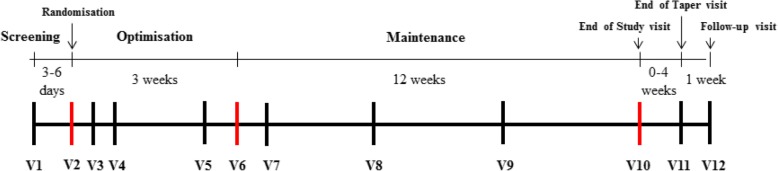
Participant timeline

During the first 3 weeks of treatment period 4 (V3–V6 in the participant timeline) (Fig. 1), visits will be scheduled to assess safety and tolerability and to titrate liquid gabapentin (75 mg/ml) to an optimal, weight-based (two weight groups, 5 to ≤ 15 kg and > 15 kg) tolerable dosage. A dose will be indicated as optimal if the subject has reached a pain intensity < 4/10 in all pain assessments in the last 48 h (*n* = 4) or the maximum tolerable dose. Only one dose reduction is allowed during the optimization period (Table 3).

**Table 3:** Dose optimization schedule of gabapentin

Weight group	V2	V3	V4	V5	V6
5–15 kg	7 mg/kg/day corresponding to 0,09 ml/kg/day	14 mg/kg/day corresponding to 0,19 ml/kg/day	21 mg/kg/day corresponding to 0,28 ml/kg/day	42 mg/kg/day corresponding to 0,56 ml/kg/day	63 mg/kg/day corresponding to 0,84 ml/kg/day
> 15 kg	5 mg/kg/day corresponding to 0,07 ml/kg/day	10 mg/kg/day corresponding to 0,13 ml/kg/day	15 mg/kg/day corresponding to 0,2 ml/kg/day	30 mg/kg/day corresponding to 0,4 ml/kg/day	45 mg/kg/day corresponding to 0,6 ml/kg/day

All children will have a titration in morphine starting at 0.6 mg/kg/day to a maximum dose of 1.2 mg/kg/day four times daily from days 1 to 5, based on the World Health Organization (WHO) guidelines of 2012 [30]. For patients with a body weight of 30 kg or less, a liquid formulation will be used during titration and maintenance phases. For patients with a body weight of 30 kg or more, a solid immediate release formulation will be used during titration phase, which will be converted to a similar daily dose solid extended release formulation during maintenance phase. During all visits in the dose optimization and maintenance period, adverse effects of morphine will be closely monitored (e.g., constipation, for which laxatives can be given).

At dose optimization visit V5 or V6 (or at end-of-study [EOS] visit V10), four venous blood samples will be taken for assessment of PK: One predosing and three at different times postdosing (four times 1.5 ml and for children less than 15 kg four times 1 ml).

#### Fixed-dose maintenance period

After dose optimization, patients will continue to take medication (gabapentin or placebo) next to morphine for an additional 12 weeks. Dose adjustments are not allowed during the maintenance period.

### End of study

For all patients who terminate or complete the dose maintenance period, medication will be tapered according to a schedule over 0–4 weeks. During this tapering period, site staff will contact the patients to ensure that they are complying with the taper schedule. At EOS visit V10, venous blood sampling will be done once again (standard clinical hematology and biochemistry [2.5 ml] and metabolomics [1 ml]).

### Study taper and follow-up period

#### End of taper

All patients who are tapered off medication will return for a visit 1–4 weeks after their EOS visit for the collection of final safety evaluations.

#### Follow-up

Seven days after the last dose of investigational medication, follow-up by phone will take place to collect information about pain intensity, global health, and ongoing or new (serious) adverse events and concomitant medication until all safety concerns are resolved.

#### Blinding

Gabapentin and placebo (oral liquid formulation) will be indistinguishable in appearance to maintain the study blinding. Also, labeling will not allow recognition of actual treatment. During the trial, blinding will be broken by the investigator for emergency purposes only, where knowledge of the blinded treatment could influence further patient care. In addition, the safety contact will unblind safety reports, as per regulatory requirements. Study blinding will be broken after database lock.

#### Efficacy measurement

The following scales will be used to assess pain intensity at all visits throughout the study. The FLACC scale is used for children between 3 months and 7 years of age who are unable to self-report their pain. It is a five-item scale that raters (investigators, parents) use to score in five categories (Face, Legs, Activity, Cry, and Consolability), each assigned a score of 0, 1, or 2 and total score between 0 and 10.

The FPS-R is a self-report measure of pain intensity. It consists of six line drawings of faces that are scored 0 to 10. In this study, the FPS-R will be used for assessment of pain in children 3 years and older because it has not been validated for younger children. In children of 3 and 4 years old who are unable to self-report using the FPS-R, the FLACC scale will be used.

The Numeric Rating Scale (NRS-11) is designed for pain score to be used by children from 8 years of age. The user has the option to verbally rate their scale from 0 to 10 or to place a mark on a line indicating their level of pain, with 0 meaning absence of pain and 10 meaning the most intense pain possible.

To be able to compare data from the different pain scores, all scores will be reported on a scale of 0–10, as indicated by the individual scores.

Pain will be assessed at the following time points:

1. Baseline assessment: average pain score of twice-daily assessment on 3 consecutive days
2. During trial: daily in the morning and when breakthrough pain is noticed
3. Endpoint: average pain score of twice-daily assessments on 3 consecutive days

Furthermore, parents and/or subjects are asked to keep a daily patient diary recording the following items: study drug intake changes, comedication intake including rescue medication, adverse events, and abnormalities in sleep.

Additionally, the following items will be scored:

- Global satisfaction with treatment (at EOS visit V10) using NRS-11
- Clinical Global Impressions Scale (CGI-EI [Investigator]) at V2, V6, and EOS V10
- Patient Global Impression of Change (PGIC) Scale at V6 and EOS V10
- Pediatric Quality of Life Inventory (PedsQL) Generic Core Scale
- Five Point Facial Hedonic Scale at EOS V10 for acceptability of the oral suspension
- Adverse events collection
- Columbia Suicide Severity Rating Scale (C-SSRS) at V1 and EOS V10
- Modified Overt Aggression Scale at V2, V6, and EOS V10

#### Outcome measurements

The primary outcome of the study is the average pain score (defined as average pain score of twice-daily assessments of 3 consecutive days) at the end of the study period in both treatment groups. Secondary endpoints are percentage of responders with 30% reduction in pain score, average daily pain intensity by age-appropriate scale, observational pain assessment with use of NRS-11 by parents/caregivers and investigator at each visit, number of episodes of breakthrough pain > 4/10 pain score and use of rescue medication, number of rescue interventions, number of pain-free days with < 4/10 pain score without rescue medication, participant dropouts due to lack of efficacy, total cumulative weight dose of each rescue drug, quality of life scored with the PedsQL at baseline (V2) and EOS visit (V10), acceptability of treatment (Facial Hedonic Scale) at EOS visit, global satisfaction with treatment (NRS-11 completed by parent/patient) at EOS visit, CGI-S at V6 and EOS (CGI-S by investigator), patient/parent PGIC at V6 and EOS, PK parameters: apparent clearance (CL/F), apparent volume of distribution (Vd/F), absorption rate constant (Ka), area under the curve (AUC), maximum (peak) concentration (Cmax), time of maximum concentration (Tmax), concentration at steady-state (Css), minimum concentration (Cmin) incidence of adverse events, percentage of adverse events, percentage of subjects discontinuing the trial due to adverse events, aggressive behavior in children > 6 years old (Retrospective-Modified Overt Aggression Scale) at V2–V6 and EOS, and suicidal ideation (C-SSRS) at V1 and EOS.

#### Study procedures

**Fig. 2:**
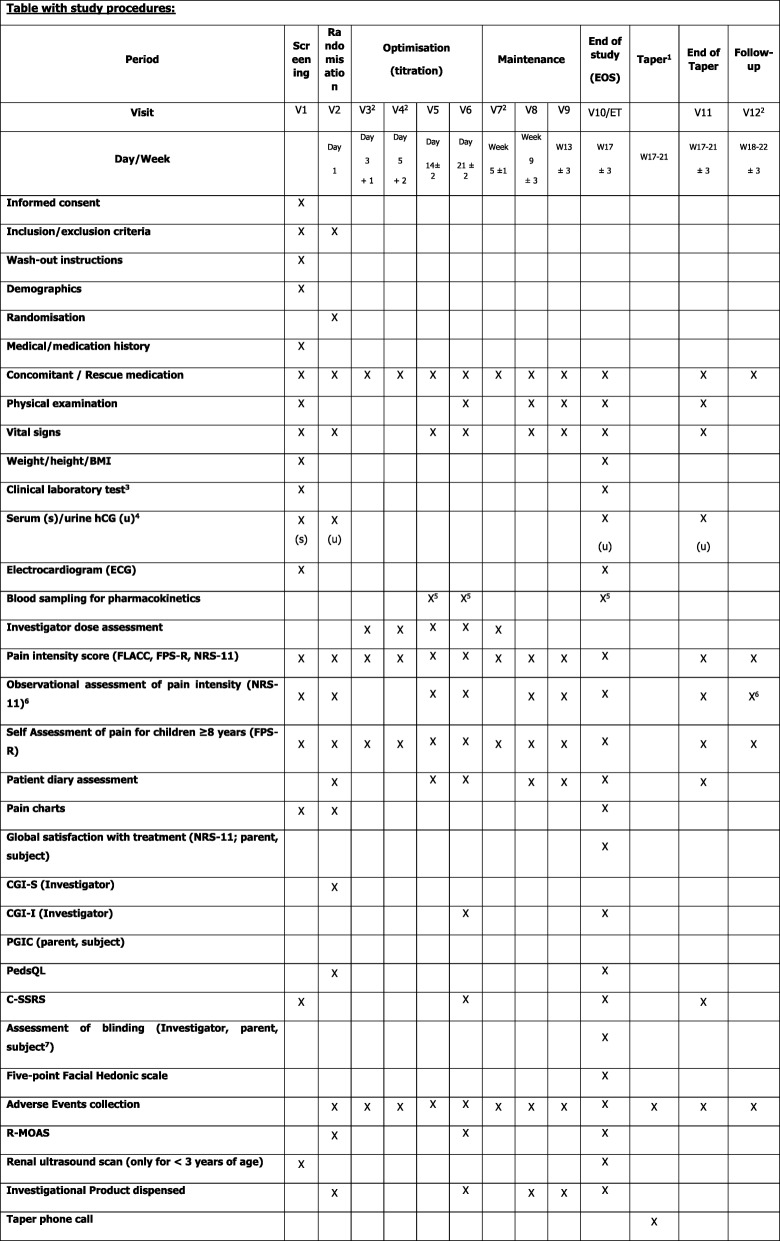
Standard Protocol Items: Recommendations for Interventional Trials (SPIRIT) figure with overview of the study procedures

The protocol structure follows the Standard Protocol Items: Recommendations for Interventional Trials (SPIRIT) 2013 statement. The SPIRIT schedule with a complete overview of the study procedures for this trial is summarized in Fig. 2. The complete SPIRIT checklist for the study is provided in Additional file 1.

### Statistical analysis

#### Sample size calculation

A total sample size of 66 is needed. It will allow detection of a significant difference between morphine plus placebo and morphine plus gabapentin groups in mean pain scores at EOS. Mean baseline pain scores of 8.5 are assumed for both groups, with estimated EOS pain scores of 5.4 for morphine alone versus 4.5 for morphine plus gabapentin based on percentage change in pain scores in a previous trial in adults [31].

A minimum number of patients is defined per age group: (1) at least 5 patients aged 3 months to less than 3 years, (2) at least 15 patients aged 3 years to less than 7 years, and (3) at least 20 patients aged 7 years to less than 18 years. No maximum number of patients per age group is specified. A two-tailed test will be applied with a significance level of 0.5 and a power of 80%, and 10% dropout is anticipated.

#### Type of analysis

The primary efficacy analysis of this study is aimed at assessing the superiority of treatment with morphine and gabapentin compared with morphine and placebo. The hypothesis to be tested is that the average pain scores (average of two measures each day for 3 days before EOS visit V10) assessed by age-appropriate pain scales (FLACC, FPS-R, NRS-11) are lower in the treatment group (morphine + gabapentin) than in the control group (morphine + placebo).

The primary analysis of efficacy will be conducted using one-way analysis of covariance (ANCOVA) with baseline average pain score as a covariate. Other covariates will include center, treatment, and age (three subgroups). A second ANCOVA model including a treatment × center interaction term will be used to assess consistency across sites.

Intergroup differences as breakthrough pain and frequency and dosage of rescue medication will be assessed using sample *t* test or Wilcoxon-Mann-Whitney tests.

Statistical analysis will be performed using IBM SPSS Statistics 21 (IBM, Armonk, NY, USA) or SAS 9.4 (SAS Institute, Inc., Cary, NC, USA) software for PC.

#### Pharmacokinetic-pharmacodynamic analysis

PK data will be analyzed by nonlinear mixed effects modeling with NONMEM (version 7.2; ICON Development Solutions, Dublin, Ireland) to estimate PK parameters, including volume distribution, clearance of gabapentin and morphine, and variability and precision. Also, influence of potential covariates will be evaluated.

Using predicted drug concentrations, a population PK-pharmacodynamic (PD) model will be developed to link drug exposure to pain response.

### Ethical approval

Ethical approval was obtained from the Medical Ethical Committee of the Erasmus Medical Centre, Rotterdam, The Netherlands.

Medical ethical review is under way in the other participating centers (EudractCT, 2014-004897-40), and the study is registered at ClinicalTrials.gov (NCT03275012).

### DSMC

The study safety and progress will be reviewed by an independent data and safety monitoring committee (DSMC) according to the DSMC charter. All adverse effects reported by the subjects or observed by investigators or staff will be recorded. A continuous evaluation will be performed by an independent DSMC. In case of disproportionate adverse effects or prolonged inclusion, the DSMC can decide to terminate the study. No interim analysis for efficacy is planned.

## Discussion

The GABA-2 study is an exploratory, randomized, double-blind, placebo-controlled, multicenter study designed to evaluate the efficacy of gabapentin added to morphine in pediatric patients with severe neuropathic or mixed pain.

Although strong opioid use for benign pain is met with cultural barriers in many countries, the use of morphine is recommended by the WHO guidelines for persistent pain in children as a first-line treatment [30]. Also, the use of morphine for NP is questioned by practitioners, but tramadol and morphine have shown efficacy in several randomized controlled trials concerning different types of NP [3–5, 32] and are therefore advised in the guidelines from the International Association for the Study of Pain for the treatment of NP in adults [32, 33].

Hence, the use of morphine for severe NP in children should be seriously considered and studied clinical trials. Because treatment with first-line agents such as antidepressants and anticonvulsants as monotherapy for severe NP is not always sufficient [34], and because data show a synergistic effect of gabapentin when added to morphine in adult patients with NP, this design was chosen for the children with the most severe pain (pain scores 7 or higher on a scale of 0–10).

Although opioids other than morphine, based on PD properties, might be more suitable [8], they were considered inappropriate for this study because of unavailability in some of the participating countries.

Chronic pain, specifically NP in children, is often difficult to treat. Children are often undertreated owing to a lack of evidence or medication is used off-label because it often is not registered for use under a certain age. For example, in The Netherlands, gabapentin is not registered for treatment of NP in patients younger than 12 years. Moreover, high-quality studies investigating the efficacy and safety of gabapentin for this indication in children are lacking. Hence, we believe our trial is necessary and timely.

Pediatric PK studies of gabapentin [35, 36] have shown that oral clearance of gabapentin was directly proportional to creatinine clearance, which is higher for children younger than 5 years of age than in older children and highly variable in infants younger than 1 year of age. Therefore, a PK analysis was performed by simulating different dosing scenarios to evaluate dosing requirements to ensure effective drug concentrations of gabapentin as described in treatment of adult NP [37]. Based on this analysis, an appropriate dosing schedule based on weight bands for practical considerations will be used.

Justification of this dosing regime will be further investigated by assessing drug exposure using sparse sampling techniques. A correlation between exposure and analgesia will be explored.

The dosing regimen for morphine and slow-release morphine is based on the WHO guideline for morphine in children [30], and it will be titrated on the basis of tolerability in the first days of the active treatment phase of the trial. Slow-release morphine with lower daily dosage would be the preferred formulation, but it is not available or registered for all age groups. Therefore, depending on the body weight of the patient as well as on availability and registration in the participating countries, immediate-release formula or tablet morphine is chosen for the titration phase. Slow-release morphine is chosen for patients above 30 kg during the maintenance period of the study.

The primary endpoint of the study will be the differences in average pain scores for the two treatment groups (average of two measures each day for 3 days before the EOS visit) assessed by the age-appropriate pain scales (FLACC, FPS-R, NRS-11). Because the age range of the study population is wide, different pain scores were needed to ensure the most optimal scale for the different age groups. FLACC is a validated, observer-based scores for younger children. For school-age children, the FPS-R was chosen as a validated self-reported pain score, whereas the NRS-11 was considered most appropriate for older children. We realize that these scores are only validated for acute pain, but in the absence of validated pain tools for chronic pain, these scores were considered the best validated alternatives. Another limitation is that the data need to be analyzed in an aggregate way (i.e., all age groups together). For this reason, we also used scores that are expressed on a 10-point scale and can be analyzed together, although we acknowledge that cross-validation of the absolute scores has not been done, to our knowledge. In general, a pain score of 4 is accepted as a cutoff for pain that needs treatment for all scores; hence, we considered the combined use of scores acceptable.

To our knowledge, this is the first randomized controlled trial studying the effectiveness of the combination therapy morphine and gabapentin for pain in children. Although very challenging in design and in potential recruitment and retention of patients, we believe this trial will provide more solid evidence of the efficacy of gabapentin in combination with morphine to ultimately improve the treatment of severe NP in children.

## Trial status

For the Gaba-2 study, ethical approval has been obtained in one of the participating centers, and the study is under review in the other centers. No patients have yet been included.

## Additional file

Additional file 1: SPIRIT 2013 checklist: recommended items to address in a clinical trial protocol and related documents. (DOC 122 kb)

## Abbreviations

ANCOVA: Analysis of covariance
CGI: Clinical Global Impressions Scale
CNS: Central nervous system
C-SSRS: Columbia Suicide Severity Rating Scale
DSMC: Data and safety monitoring committee
EOS: End of study
FLACC: Face, Legs, Activity, Cry, Consolability scale
FPS-R: Faces Pain Scale–Revised
GAPP: Gabapentin in Paediatric Pain
NP: Neuropathic pain
NRS-11: Numeric Rating Scale
NSAID: Nonsteroidal anti-inflammatory drug
PD: Pharmacodynamic
PedsQL: Pediatric Quality of Life Inventory
PGIC: Patient Global Impression of Change Scale
PIP: Pediatric investigation plan
PK: Pharmacokinetic(s)
SPIRIT: Standard Protocol Items: Recommendations for Interventional Trials
WHO: World Health Organization
