# Correction to: Gabapentin as add-on to morphine for severe neuropathic or mixed pain in children from age 3 months to 18 years - evaluation of the safety, pharmacokinetics, and efficacy of a new gabapentin liquid formulation: study protocol for a randomized controlled trial

**DOI:** 10.1186/s13063-019-3519-9

**Published:** 2019-06-20

**Authors:** Thomas G. de Leeuw, Laura Mangiarini, Rebecca Lundin, Florentia Kaguelidou, Tjitske van der Zanden, Oscar Della Pasqua, Dick Tibboel, Adriana Ceci, Saskia N. de Wildt, on behalf of the GAPP consortium

**Affiliations:** 1 Department of Anesthesia and Pain Medicine, Erasmus MC-Sophia Children’s Hospital, Dr. Molewaterplein 40, 3015 GD Rotterdam, The Netherlands; 2 Consorzio per Valutazione Biologiche e Farmacologiche, Pavia, Italy; 3 Fondazione Penta Onlus, Padova, Italy; 4 Department of Pediatric Pharmacology and Pharmacogenetics, AP-HP, Hôpital Robert Debré, F-75019 Paris, France; 5 Inserm, CIC 1426, F-75019 Paris, France; 6 Université Paris Diderot, Sorbonne Paris Cité, EA 08, F-75010 Paris, France; 7 Intensive Care and Department of Paediatric Surgery, Erasmus MC-Sophia Children’s Hospital, Dr. Molewaterplein, 40 3015 GD Rotterdam, The Netherlands; 8 University College London, London, UK; 9 Department of Pharmacology and Toxicology, Radboud University, Nijmegen, The Netherlands

**Correction to: Trials (2019) 20:49.**

**https://doi.org/10.1186/s13063-018-3169-3**

Following publication of the original article [1], we have been notified that the tagging of one of the author names was done incorrectly in the XML version of the paper. Original and corrected tagging can be seen below.

- Incorrect name tagging:
  Last Name: Pasqua.
  First Name: Oscar Della.
- Correct author name tagging:
  Last Name: Della Pasqua.
  First Name: Oscar.
